# Detection of Nipah and Hendra Viruses Using Recombinant Human Ephrin B2 Capture Virus in Immunoassays

**DOI:** 10.3390/v14081657

**Published:** 2022-07-28

**Authors:** Ming Yang, Wenjun Zhu, Thang Truong, Bradley Pickering, Shawn Babiuk, Darwyn Kobasa, Logan Banadyga

**Affiliations:** 1 National Centre for Foreign Animal Disease, Canadian Food Inspection Agency, Winnipeg, MB R3E 3M4, Canada; wenjun.zhu@inspection.gc.ca (W.Z.); bradley.pickering@inspection.gc.ca (B.P.); shawn.babiuk@inspection.gc.ca (S.B.); 2 National Microbiology Laboratory, Public Health Agency of Canada, Winnipeg, MB R3E 3R2, Canada; thang.truong@phac-aspc.gc.ca (T.T.); darwyn.kobasa@phac-aspc.gc.ca (D.K.); logan.banadyga@phac-aspc.gc.ca (L.B.); 3 Department of Medical Microbiology and Infectious Diseases, University of Manitoba, Winnipeg, MB R3E 0J9, Canada; 4 Department of Veterinary Microbiology and Preventative Medicine, College of Veterinary Medicine, Iowa State University, Ames, IA 50011, USA

**Keywords:** henipavirus, nipah virus, hendra virus, glycoprotein, receptor, ephrin B2, immunoassay, ELISA, lateral flow strip test

## Abstract

Nipah virus (NiV) and Hendra virus (HeV) are classified as high-consequence zoonotic viruses characterized by high pathogenicity and high mortality in animals and humans. Rapid diagnosis is essential to containing the outbreak. In this study, the henipavirus receptor ephrin B2 was examined to determine whether it could be used as a universal ligand for henipavirus detection in immunoassays. Enzyme-linked immunosorbent assays (ELISAs) were developed using recombinant ephrin B2 as the capture ligand and two monoclonal antibodies (mAbs) as detection reagents. Using mAb F27NiV-34, which cross-reacts with NiV and HeV, we were able to detect NiV and HeV, while mAb F20NiV-65 was used to detect NiV. Therefore, using these two ELISAs, we were able to differentiate between NiV and HeV. Furthermore, we developed a rapid lateral flow strip test for NiV detection using ephrin B2 as the capture ligand combined with mAb F20NiV-65 as the detector. Taken together, our results show that the combination of ephrin B2 and a specific mAb provides an excellent pairing for NiV and HeV detection.

## 1. Introduction

Nipah virus (NiV) and Hendra virus (HeV) are classified as high-consequence zoonotic viruses and are characterized by high pathogenicity and high mortality in animals and humans [1,2,3]. Both NiV and HeV are classified as containment level 4 (CL4) pathogens and NiV is currently listed as one of the World Health Organization (WHO)’s priority diseases that pose the greatest public health risk due to their epidemic potential, high lethality, and lack of therapeutics for clinical treatment [2].

HeV was first described in 1994 during an outbreak of the respiratory and neurologic disease in horses and humans in Hendra, Australia. Since its emergence, HeV continues to pose a serious public health threat to the equine and veterinary industries. HeV causes sporadic disease outbreaks in Northeastern Australia. There were around 53 incidents associated with HeV that have been reported since July 2016 [2,4]. The first NiV outbreak occurred in Malaysia in 1998 and was responsible for the fatal encephalitis that resulted in the deaths of humans and the culling of pigs, which caused extensive economic loss for the local swine industry [5]. Since then, NiV causes disease outbreaks in Southeast Asia almost every year. Five countries in South and Southeast Asia, Malaysia, Singapore, Bangladesh, India, and the Philippines have all been affected by NiV. The latest report of NiV infection has been recorded in September 2021 from Kerala, India which is the fifth outbreak of the disease in India [6,7,8]. NiV strains circulating in Bangladesh and India (Clade I, or NiV-B) are different from the strains identified in Malaysia and Singapore (Clade II, or NiV-M) [1,2,9,10,11].

NiV and HeV belong to the family *Paramyxoviridae*, under the genus *Henipavirus.* Like all other viruses in the subfamily *Paramyxovirinae*, the genomes of NiV and HeV are non-segmented, single-stranded negative-sense RNA [12]. Henipaviruses contain six transcription gene units encoding six major structural proteins, namely the nucleocapsid protein (N), phosphoprotein (P), matrix protein (M), fusion protein (F), glycoprotein (G), and large protein or RNA polymerase, and three predicted non-structural proteins: C, V, and W [2,13,14].

Ephrin B2 and B3 have been identified as functional cellular receptors for henipaviruses, with a high affinity for the henipavirus surface glycoprotein G [15,16,17]. Ephrin B2 is expressed on neurons, endothelial cells, the smooth muscle surrounding arteries, placental tissue, spleen, and sinusoidal lining of lymph nodes [18]. Ephrin B3 is expressed on lymphoid cells, which may account for the NiV infection-induced acute lymphoid necrosis [16,19]. The broad species tropism of henipaviruses is largely due to the protein sequence conservation of ephrin B2 and B3 across many species [20].

There is a licensed HeV vaccine for horses in Australia, however, there are currently no NiV and HeV vaccines licensed for use in humans [1,21]. As such, early detection of NiV and HeV infections is critical during an outbreak to identify cases and limit the spread of the viruses. Previously, Bossart et al. [22] reported the development of an assay for the detection of anti-NiV and HeV antibodies using soluble glycoproteins and their receptor ephrin B2, in a multiplexed microsphere platform. Using a virus receptor to capture viruses for the detection of foot-and-mouth disease virus (FMDV) has been reported [23,24]. In this study, we report that the henipavirus receptor, recombinant ephrin B2 can be used as a ligand to capture viruses for the detection of NiV and HeV in enzyme-linked immunosorbent assay (ELISA) and lateral flow immunochromatography (LFI) strip tests. The combination of ephrin B2 and specific monoclonal antibodies (mAbs) provides an excellent pairing for NiV and HeV detection. These findings provide valuable information for the development of future immunoassays with the goal of accurate and rapid detection of NiV and HeV.

## 2. Materials and Methods

### 2.1. Preparation and Inactivation of Nipah and Hendra Viruses

The viruses used in this study, NiV-M (Accession No. AF212302), NiV-B (Accession No. AY988601.1), and HeV (Accession No. NC_001906.3) were provided by the Centers for Disease Control and Prevention (CDC) in Atlanta, Georgia and the Public Health Agency of Canada (PHAC) in Winnipeg, Manitoba. The virus preparation procedure was described previously by Berhane et al. [25]. Briefly, African green monkey kidney (Vero-76) cells (ATCC) were infected with NiV or HeV. Viruses were harvested 24 h post-infection and clarified by centrifugation at 8000× *g* for 30 min. Virus titers were determined using virus plaque assays [26]. The plaque-forming units are 8.12 × 10^6^ plaque-forming units (pfu)/mL for NiV-B, 8.25 × 10^6^ pfu/mL NiV-M, and 4.44 × 10^7^ pfu/mL for HeV. Viruses were inactivated using 10 mM 2-Bromoethylamine Hydrobromide (BEI, Sigma-Aldrich, St Lucia, MO, USA) [25] and ready for use in ELISA and LFI strip tests. For immunization, the inactivated virus was concentrated through a 20% sucrose cushion using the Beckman SW-28 rotor at 28,000 rpm for 2 h at 4 °C. The resuspended pellet was layered onto a 30–60% discontinuous sucrose-density gradient and centrifuged using an ultracentrifuge (Beckman Optima LE-100K). The bands collected from the sucrose gradient were collected for mouse immunization.

### 2.2. Recombinant Protein Expression and Purification

Full-length NiV-B glycoprotein (G) lacking the cytomembrane and transmembrane domains (GenBank: AY988601.1) was synthesized with codon optimization and cloned into pAB-bee™ –FH vector containing a polyhedron promoter (AB Vector, LLC, San Diego, CA, USA) by GenScript (GenScript USA Inc., Piscataway, NJ, USA). The purified pAB-bee™ –FH vector containing the NiV-G gene was co-transfected with linearized baculovirus vector DNA, ProFold™-ER1 (AB Vector, LLC, San Diego, CA, USA) into Sf9 insect cells (Expression System) to generate a recombinant baculovirus containing a NiV-G gene. The recombinant baculovirus was plaque purified. A single clone was subcultured on Sf9 cells at 27 °C with shaking at 130 rpm for 120 h and subsequently used to infect suspension cultures of *Trichoplusia ni* (Tni) cells (Expression Systems) at a multiplicity of infection (MOI) of 5 to 10. The infected cells were harvested 72 h post-infection. Recombinant NiV-G was purified using Ni-NTA resin (Qiagen, Germantown, MD, USA), and the final product was confirmed by Western blot.

Soluble form of His-tagged recombinant human ephrin B2 was expressed and purified by GenScript Inc. (Piscataway, NJ, USA). Briefly, ephrin B2 (GenBank: NP_004084 (1-227)) was synthesized with codon optimization and was cloned into a pcDNA3.4 vector at the Cloning Sites of EcoRI (G/AATTC)/HindIII (A/AGCTT) and fused with His-tag at C-terminal. The purified pcDNA3.4 vector containing the ephrin B2 gene was transfected into the HD 293F cell line using a High Density (HD) Transient Expression system, the supernatant was collected, and then the His-tagged ephrin E2 protein was purified by one-step purification using an affinity column.

### 2.3. Western Blot Analysis of Recombinant NiV-G and Ephrin B2

Recombinant NiV-G and human ephrin B2 proteins were separated with 10% NuPAGE Novex Bis-Tris gels (Invitrogen, Carlsbad, CA, USA) followed by protein transfer to nitrocellulose (NC) membranes using the iBlot Gel Transfer Device (Invitrogen, Carlsbad, CA, USA). Membranes were blotted in PBS–T with 5% skim milk at room temperature for 1 h and then incubated with the following antibodies: a horseradish peroxidase (HRP)-conjugated mAb against the His-tag (EMDMillipore, Burlington, MA, USA), a polyclonal rabbit anti-NiV-G antibody (CD Creative Diagnostics, New York, NY, USA), or an anti-ephrin B2 mAb (1:2000, cat # sc-398735, SANTA CRUZ Biotechnology, Inc. Dallas, TX, USA). Following primary antibody incubation, blots were incubated with an HRP–conjugated anti-rabbit or anti-mouse secondary antibody (1:2000; Jackson ImmunoResearch Laboratories, West Grove, PA, USA) for 1 h at room temperature. Blots were developed using the substrate 3,3′–Diaminobenzidine (Sigma-Aldrich, St. Louis, MO, USA).

### 2.4. Generation of Monoclonal Antibodies

Mouse immunization and fusion were performed as previously described [25]. Briefly, female Balb/c mice were inoculated subcutaneously with purified NiV-M (~20 µg/mouse) in an equal volume of complete Freund’s adjuvant and incomplete Freund’s adjuvant (Difco, BD, Oakville, ON, Canada). Three identical immunizations were administered at four-week intervals with a final boost prior to fusion. Spleen cells from immunized mice were fused with myeloma cells (P3 × 63 Ag8.653, ATCC, Rockville, MD, USA). Hybridoma supernatants were screened using indirect ELISA. The positive clones were subcloned using a limiting dilution method. The mAbs were isotyped using a mouse mAb isotyping kit (Roche, Indianapolis, IN, USA).

### 2.5. Indirect ELISA

Microtitre plates (Nunc-Immunoplate, Roskilde, Denmark) were coated with recombinant NiV-G, NiV-N (Cat# REC31746, NativeAntigen Company, Kidlington, UK) or Ebola virus glycoprotein (EBOV-GP, obtained from PHAC) in a carbonate/bicarbonate buffer, pH 9.6 overnight at 4 °C. Following blocking with Casein blocking buffer (Sigma-Aldrich, USA), mAbs in casein blocking buffer were added. After incubation, HRP–conjugated anti-mouse IgG (1:2K, Jackson ImmunoResearch Laboratories, West Grove, PA, USA) was added. Then 3,3′,5,5′-Tetramethylbenzidine (TMB, Pierce Biotechnology, Inc. Rockford, IL, USA) was added. After stopping, optical density (OD) was measured at 450 nm using an Emax microplate reader (Molecular Devices, San Jose, CA, USA). Each incubation step was 60 min at 37 °C with gentle shaking followed by washing five times with a washing buffer (PBS with 0.1% Tween 20).

### 2.6. Sandwich ELISA

Microtitre plates (Nunc-Immunoplate Maxisorp, Roskilde, Denmark) were coated with rabbit poly anti-NiV-G (1:2K, Creative Diagnostics, USA) or recombinant ephrin B2, or B3 (25 ng/well, Biotechne, Minneapolis, MN, USA) in a carbonate/bicarbonate buffer at a pH 9.6 overnight at 4 °C. Following blocking with casein blocking buffer, inactivated viruses (NiV-B, NiV-M, HeV or FMDV) were added to the plates, and then mAbs (hybridoma culture supernatants, 1:20) or polyclonal sera (1:500) [27] diluted in casein blocking buffer were added. After incubation, HRP-conjugated anti-mouse or anti-pig IgG (Jackson ImmunoResearch Laboratories, West Grove, PA, USA) were added. The color development process is the same as described above for the indirect ELISA.

### 2.7. Plaque Reduction Neutralization Tests

Plaque Reduction Neutralization Tests (PRNT) were carried out under CL4 containment and performed as previously described [25].

### 2.8. Biotinylation of the Recombinant Human Ephrin B2

Ephrin B2 biotin conjugation is performed using the BiotinTag Micro Biotinylation Kit (Sigma-Aldrich, USA). BAC- Sulfo N-hydroxysulfosuccinimide (NHS) was dissolved using 30 µL dimethylsulfoxide (DMSO) and then 0.1 M PBS was added to a final concentration of 5 mg/mL. The BAC-Sulfo-NHS (10 µL) was added to the recombinant human ephrin B2 (1 mg) in 0.1 M PBS and incubated for 30 min at room temperature. Following incubation, the unbound biotin was removed by extensive dialysis against PBS at 4 °C. The reagent was stored at 4 °C in PBS with 0.01% NaN_3_.

### 2.9. Purification and Colloidal Gold Conjugation of the Monoclonal Antibody

Monoclonal antibody purification and gold conjugation were performed as previously described [28]. Briefly, hybridoma culture supernatants were purified using a HiTrap Protein-G affinity column (GE, Fairfield, CT, USA) in an AKIA chromatography system.

The purified mAb was gold conjugated using the High Sensitivity Conjugation kit (80-nm Gold Nanospheres, nanoComposix, Inc., San Diego, CA, USA). Briefly, 70 µg 1-Ethyl-3-(3-dimethylaminopropyl) carbodiimide (EDC) and 140 µg Sulfo-NHS were added to 1 mL Gold Nanospheres and incubated for 30 min at room temperature to activate carboxyl gold. The gold solution was washed twice with a 1 mL potassium phosphate reaction buffer (pH 7.4). The purified mAb (20 µg) was added to the gold solution and incubated for 1 h, followed by washing twice with a reaction buffer. The gold conjugated mAb was re-suspended in the conjugation buffer (PBS with 0.5% BSA, 0.5% casein, 1% tween 20, and 0.05% azide).

### 2.10. Lateral Flow Immunochromatographic Strip Test

The rapid assay device (gRAD) used in this study was purchased from Bioporto Diagnostics A/S (Copenhagen, Denmark). The test principle was described in a previous report [29]. Briefly, there are two lines on the gRAD strip: A test line containing a biotin-binding protein, and a control line containing an anti-mouse antibody. When testing, inactivated NiV or HeV in cell culture supernatants (25 µL) were mixed with the biotin-conjugated recombinant ephrin B2 (0.25 µL/per test), and the gold-conjugated detection mAb F20NiV-65 (2 µL/per test) to form a complex in PBS (25 µL) and running buffer (50 µL, 1% Tween 20 in PBS). The strip was dipped into each tube containing the mixture of sample/ephrin B2/antibodies. After 30 min, results were determined by visualization.

## 3. Results

### 3.1. Production and Characterization of Monoclonal Antibodies against Nipah Virus

Following mouse immunization with the BEI-inactivated NiV-M virus, splenocytes were harvested and fused with myeloma cells. Supernatant from the resulting hybridomas was screened using NiV-M as the antigen in an ELISA, positive clones were subcloned, and mAbs were isolated.

**Figure 1:**
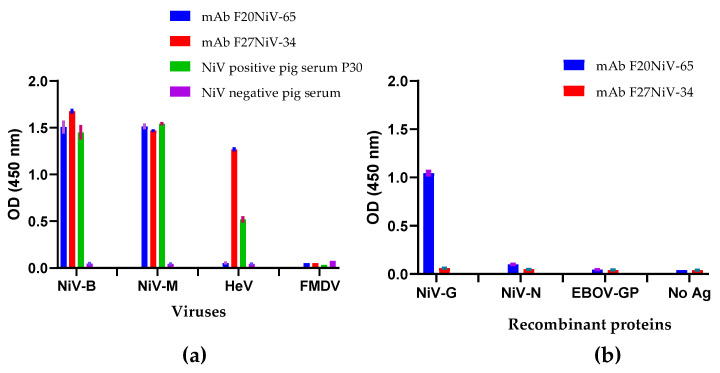
Monoclonal antibody reactivity to Nipah, Hendra virus in sandwich ELISA and reactivity to recombinant Nipah proteins in indirect ELISA. (**a**) Rabbit polyclonal anti-NiV-G antibody was coated onto microtiter plates. NiV, HeV, and foot-and-mouth disease virus (FMDV) were added to the plates. Then two monoclonal antibodies (mAbs), F20NiV-65, F27NiV-34, positive control sera (from a NiV-infected pig), and negative control sera (from a naïve pig) were added. The antibody binding was detected with either HRP-conjugated goat anti-mouse or anti-swine IgG antibodies. OD values are the mean values subtracted from the virus-free background. (**b**) Purified recombinant NiV-G, NiV-N, or EBOV-GP (negative control) were coated onto ELISA plates. Then two mAbs, F20NiV-65 or F27NiV-34, were added, and binding was detected with HRP-conjugated goat anti-mouse IgG antibody. The data shown are the mean of duplicates with error bars.

To demonstrate mAb reactivity to NiV and/or HeV, polyclonal anti-NiV-G antibodies were coated to capture inactivated NiV and HeV. FMDV was used as a negative control in sandwich ELISA. Ideally, another paramyxovirus would be used as a negative control instead of FMDV. FMDV was used in this study as no other viruses were available in our laboratory. Two mAbs, F20NiV-65 and F27NiV-34, showed reactivity to both NiV-M and NiV-B. F27NiV-34 also reacted strongly with HeV, while F20NiV-65 failed to react with HeV in the ELISA (Figure 1a). Both mAbs showed no cross-reactivity to the unrelated FMDV. Notably, F20NiV-65 demonstrated in vitro neutralization activity against NiV (PRNT titer ≥ 1:320), while F27NiV-34 exhibited weak neutralizing activity (PRNT titer ≥ 1:20). Neutralizing activity against HeV was not examined. F20NiV-65 was determined to be of the IgG2b isotype, while F27NiV-34 was determined to be IgG1; both mAbs contain ƙ light chains.

Recombinant NiV-G was analyzed by Western blot analysis prior to its use, which revealed a protein band with a predicted molecular weight of approximately 70 kDa (Figure S1). The reactivity of the mAbs to recombinant NiV-G, and NiV-N was examined using an indirect ELISA. F20NiV-65 reacted with NiV-G protein, but not with NiV-N or negative control, EBOV-GP, while F27NiV-34 did not react with any protein (Figure 1b). These results suggest that the binding site of F20NiV-65 is located on NiV-G, but the binding location of mAb F27NiV-34 remains unknown. The binding epitopes of the two mAbs are presumably conformational because neither reacted with reduced NiV protein in a Western blot. 

### 3.2. Expression of Recombinant Ephrin B2 and Selection of Ephrin B2 or B3 as Better Capture Ligand

**Figure 2:**
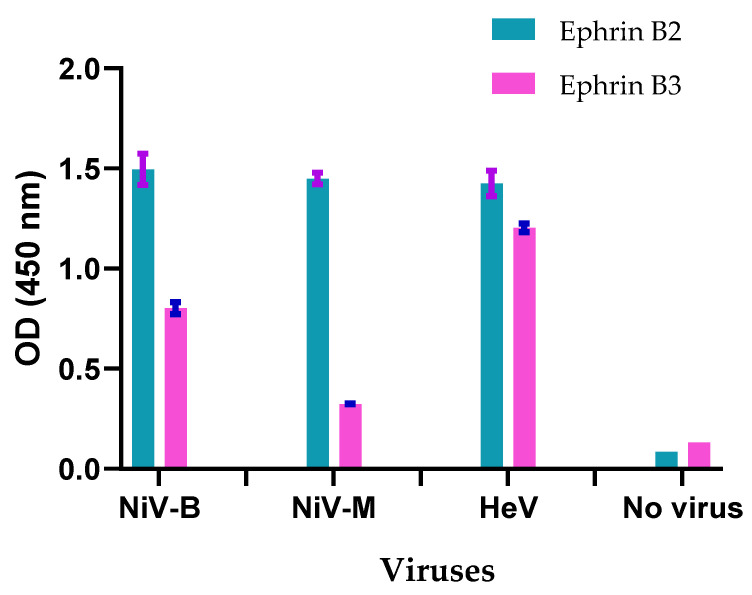
Comparison of the capture capacity of ephrin B2 and B3 for Nipah and Hendra viruses using sandwich ELISA. Recombinant ephrin B2 and B3 protein were coated onto ELISA plates. Nipah virus (NiV) strain Bangladesh (NiV-B), NiV strain Malaysia (NiV-M), or Hendra virus (HeV) were added, and then virus binding was detected using a NiV-positive pig serum. HRP-conjugated anti-swine IgG and a substrate TMB were used for color development. The data shown are the mean of duplicates with error bars.

The recombinant human ephrin B2 protein was analyzed by Western blot. A band of approximately 40 kDa corresponding to the predicted molecular weight of ephrin B2 was detected using both anti-His and anti-ephrin mAbs (Supplementary Figure S2). To determine whether ephrin B2 or ephrin B3 functions as a better capture ligand for henipavirus detection, a series of ELISAs were performed. Plates were coated with recombinant human ephrin B2 or B3. NiV and HeV were added, and virus binding to ephrin B2 or B3 was quantified using NiV-positive pig sera. The ELISA results showed that ephrin B2 had higher binding activity than ephrin B3 for NiV-B and -M, and both had similar binding affinities to HeV (Figure 2). Thus, ephrin B2 was selected as the capture ligand for henipavirus detection.

**Figure 3:**
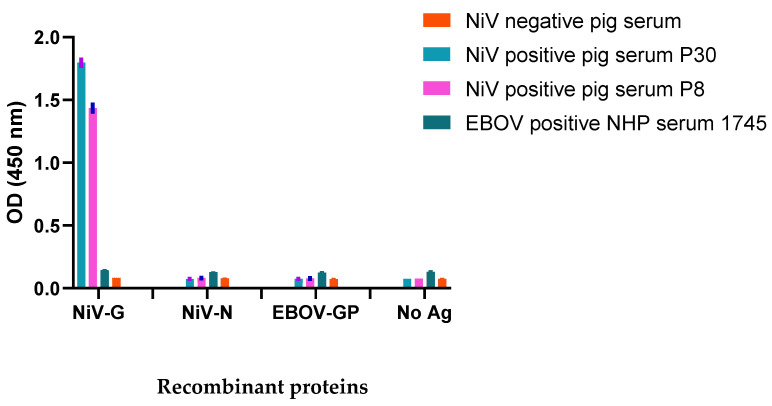
Reactivity of ephrin B2 with Nipah virus structural proteins in sandwich ELISA. Recombinant ephrin B2 was coated onto ELISA plates. Then recombinant Nipah virus (NiV) glycoprotein (G), nucleocapsid protein (N), or Ebola virus glycoprotein (EBOV-GP) were added. Binding to the protein was detected using two NiV-positive sera (pig 30, pig 8), one EBOV-positive serum (NHP), and a negative pig serum. Then HRP-conjugated anti-swine IgG or anti-NHP IgG were added. TMB substrate was used for color development. The data shown are the mean of duplicates with error bars.

To confirm that ephrin B2 captures henipaviruses by interacting with NiV-G, a second ELISA was performed. Plates were coated with ephrin B2 followed by recombinant NiV-G, NiV-N, or EBOV-GP. Since only NiV-G (not NiV-N and EBOV-GP) is expected to be captured by ephrin B2, NiV-positive pig serum should only react with NiV-G. The ELISA results showed that NiV-positive pig sera detected NiV-G, but not NiV-N and EBOV-GP, while EBOV-positive sera did not detect EBOV-GP (Figure 3), suggesting that ephrin B2 captures henipavirus by interacting with NiV-G.

### 3.3. Development of an Antigen Capture Sandwich ELISA for Nipah and Hendra Virus Detection

**Figure 4:**
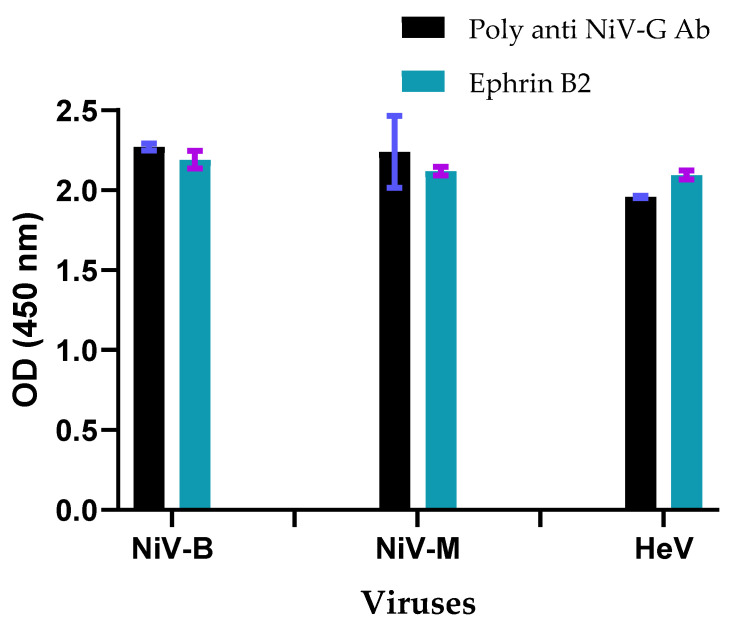
Comparison of ephrin B2 and polyclonal NiV-G for capture of Henipavirus in sandwich ELISA. Polyclonal anti-NiV-G and recombinant ephrin B2 were coated onto an ELISA plate. Inactivated NiV and HeV were added, and then viruses were detected using mAb F27NiV-34. Optical density (OD) values were the mean values subtracted from the virus-free background with error bars.

In most antigen detection ELISAs, polyclonal antibodies are used as the capture ligand. To determine whether recombinant ephrin B2 could act similarly to polyclonal anti-NiV-G antibodies as a capture ligand in a sandwich ELISA, ephrin B2 and polyclonal anti-NiV-G were coated onto separate plates. Both inactivated NiV and HeV were detected using the mAb F27NiV-34, which reacts to both NiV and HeV. The ELISA results showed similar detection using both polyclonal NiV-G- and ephrin B2-coated plates, indicating that ephrin B2 and polyclonal anti-NiV-G antibodies have the same capture ability (Figure 4).

**Figure 5:**
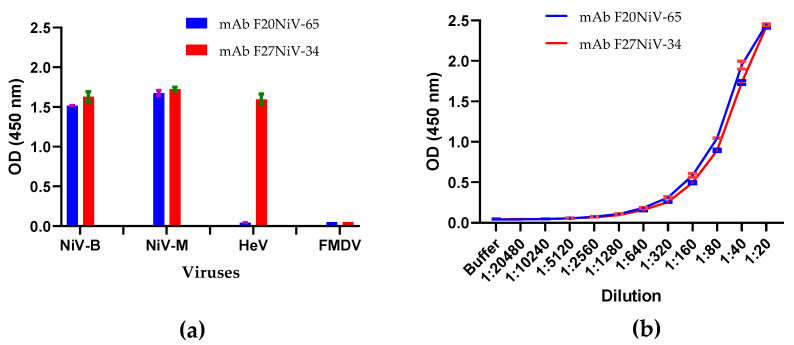
Antigen capture ELISA for Nipah and Hendra virus detection. Recombinant ephrin B2 protein was coated onto ELISA plates. After blocking: (**a**) Nipah virus (NiV) strain Bangladesh (NiV-B), NiV strain Malaysia (NiV-M), Hendra virus (HeV), or foot-and-mouth disease virus (FMDV; negative control) were added, and then viruses were detected using mAb F20NiV-65 for NiV only and F27NiV-34 for henipaviruses. HRP-conjugated anti-mouse IgG and a substrate TMB were used for color development. (**b**) Two-fold dilutions of NiV-M and HeV were added to the plate and the virus was detected using mAbs F20NiV-65 for NiV and F27NiV-34 for HeV. HRP-conjugated anti-mouse IgG and a substrate TMB were added for color development. The data shown are the mean of duplicates. The data shown are the mean of duplicates with error bars.

Given that ephrin B2 can replace polyclonal antibodies as a ligand in ELISAs to detect henipavirus, we next sought to establish a more specific antigen detection ELISA using mAbs F27NiV-34 and F20NiV-65. Since mAb F27NiV-34 reacts with both NiV and HeV, it was used to detect both henipaviruses, while mAb F20NiV-65, which reacts only with NiV, was used to detect NiV only. Using these two ELISAs, we can differentiate between NiV and HeV. The results showed that both NiV and HeV were detected in the ELISA using mAb F27NiV-34, whereas only NiV was detected using mAb F20NiV-65 (Figure 5a). No reactivity was observed against FMDV, suggesting that the assay is specific for NiV and HeV. To demonstrate a dose-dependent increase in NiV and HeV specific binding, two-fold dilutions of NiV-M and HeV were tested in antigen capture ELISAs, using mAbs F20NiV-65 for NiV and F27NiV-34 for HeV. The results showed that ELISA detected specific NiV and HeV in a dose-dependent manner (Figure 5b). The current results suggest that a NiV antigen detection ELISA using ephrin B2 as the capture ligand is a promising platform for henipavirus detection. However, full validation of this ELISA is required.

### 3.4. Ephrin B2 as a Ligand in Lateral Flow Immunochromatographic Strip Tests for Nipah Virus Detection

**Figure 6:**
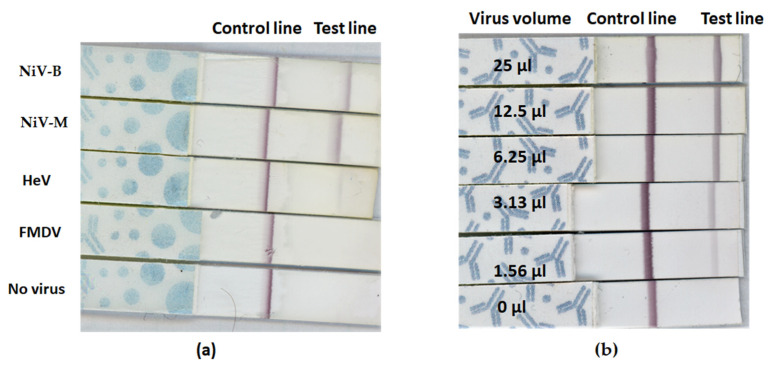
Lateral flow strip test for Nipah virus detection. The biotin-binding protein was sprayed on the test line that captures biotinylated ephrin B2. An antibody that binds the mouse antibody was sprayed on the control line. Nipah virus (NiV) strain Bangladesh (NiV-B), NiV strain Malaysia (NiV M), Hendra virus (HeV), or foot-and-mouth disease virus (FMDV) were mixed in the running buffer with Biotin-ephrin B2 and Colloidal-gold-conjugated mAb F20NiV-65. gRAD strips were dipped into the tube containing mixtures. After 15 min, results were determined by eye. A positive result is demonstrated by reddish-purple bands at both test and control lines. A negative result is demonstrated by a single visible band at the control line only. (**a**) NiV-B, NiV-M, HeV, and negative controls (FMDV or no virus) were tested using the NiV-LFI strip test, and (**b**) NiV-B (8.12 × 10^6^ plaque-forming units/mL) in the culture supernatant was 2-fold diluted in PBS and viruses were tested using the NiV-LFI strip test.

Next, we examined the ability of ephrin B2 to capture NiV in an LFI strip test, also known as a rapid strip test. Recombinant human ephrin B2 was biotin-conjugated as the capture ligand, and the two NiV-specific mAbs, F20NiV-65 and F27NiV-34, were purified and conjugated to 80-nm gold nanospheres as the detection antibodies. Using ready-made gRAD test strips, the biotin-binding protein was applied to the test line to capture biotinylated ephrin B2, while an anti-mouse antibody was applied to the control line. BEI inactivated culture supernatants containing NiV-B (8.12 × 10^6^ pfu/mL), NiV-M (8.25 × 10^6^ pfu/mL), HeV (4.44 × 10^7^ pfu/mL), or FMDV were mixed with biotinylated ephrin B2 and either gold-conjugated F20NiV-65 or F27NiV-34. When the gRAD test strip is dipped into a tube containing this mixture, a positive result is indicated when the ephrin B2/virus/mAb complex reacts with the immobilized biotin-binding protein on the test line to make the test line visible. The validity of the test is confirmed when excess gold-conjugated detection mAb binds to the immobilized anti-mouse antibody to form the control line. When mAb F27NiV-34 was used as the detection mAb, no visible test line was observed in the gRAD test strip (data not shown), despite the fact that this antibody reacted to NiV and HeV-G in earlier ELISAs. In contrast, F20NiV-65, which is specific for NiV-G, produces strong bands on the test line when detecting NiV-B and NiV-M but not FMDV (Figure 6a). Interestingly, F20NiV-65 also produced a very faint band when HeV was assayed, although the response to HeV in the ELISA assay was negative.

To confirm that the positive results with the F20NiV-65 LFI strip test were NiV-specific, culture supernatant containing NiV-B was two-fold serial diluted in PBS and tested again using the strip test. The results showed that the test line intensity decreased from strong to weak as the dilution of the test sample increased, confirming a correlation between NiV load and test result (Figure 6b). Together, these results demonstrate that ephrin B2 can be used as a capture ligand for NiV detection in a rapid LFI strip test.

## 4. Discussion

In the current study, we evaluated the use of recombinant human ephrin B2 as a common capture ligand for NiV and HeV detection in ELISA and rapid LFI strip tests. In addition, NiV-specific mAbs were generated and characterized. We show that the combination of recombinant ephrin B2 and mAbs F20NiV-65 or F27NiV-34 can be used as reagents for the detection of henipaviruses in immunoassays. F20NiV-65 and F27NiV-34 both reacted with NiV-M and -B, while F27NiV-34 also reacted with HeV. Both mAbs failed to detect NiV proteins in a Western blot analysis, indicating that their binding epitopes are conformational. The F20NiV-65 epitope is located in the NiV-G protein, as shown in the indirect ELISA; however, the F27NiV-34 epitope remains unknown because it neither reacted with recombinant NiV-G nor with NiV-N. PRNT showed that both mAbs inhibited NiV infection of target cells; inhibition by F20NiV-65 was strong, while inhibition by F27NiV-34 was weak. It has been reported that the two surface henipavirus glycoproteins, F and G, which facilitate virus attachment and entry into cells, also induce neutralizing antibodies [30]. Indeed, a critical neutralization epitope on the surface of F has been identified [31]. Since F27NiV-34 does not bind to NiV-G, we hypothesize that this mAb instead reacts to F protein. If this is the case, the high degree of sequence identity among henipavirus F [1,14,32] may explain why F27NiV-34 was able to cross-react with both NiV and HeV. The high level of cross-reactivity of this mAb with HeV-G and NiV-G is valuable for use during screening for Henipavirus infection. While it remains unclear whether these two mAbs have any prophylactic or therapeutic potential in patients with henipavirus infection, we were able to establish both antibodies as good candidates for use in diagnostic immunoassays.

NiV and HeV use host cell membrane ephrin proteins as virus entry receptors, and both bind to ephrin via G [15,16]. Although there are three ephrin B-classes (B1-B3), only ephrin B2 and ephrin B3, which share about 40% sequence identity, can serve as functional receptors for henipaviruses [18,33]. The binding pocket in Henipavirus-G that interacts with the G-H loops of ephrin B2 and B3 is highly conserved [34], but the binding affinities are unclear [33]. EphrinB2 is reported to be the primary receptor for NiV, and ephrinB3 is used as an alternate receptor, possibly accounting for some of the central nervous system lesions seen in NiV infection [35]. More recently, the affinities of ephrin-B2 and ephrin-B3 for the NiV attachment glycoprotein were characterized, and when compared, ephrin-B3 had a lower affinity for the attachment glycoprotein but still permitted virus entry [17]. To determine which of ephrin B2 or B3 is a better ligand for henipavirus detection, both recombinant humans ephrin B2 and -B3 were tested using a sandwich ELISA. The results showed that ephrin B3 binds less strongly to NiV compared to B2, while ephrin B2 and B3 showed similar binding to HeV. Our results disagree with two previously published reports. One report using recombinant NiV-G suggested NiV-G and HeV-G bound to human ephrinB2 with similar affinities, NiV-G bound ephrinB3 with a ~30-fold higher affinity than that of HeV-G [35]. Another report indicated that HeV-G appears to have a lower affinity for ephrin B2 than NiV-G [36]. There may be many factors that affect the binding of ephrin B2 to henipavirus glycoproteins, such as reaction buffers, binding conditions, and recombinant proteins expressed in different systems. In addition to the above-mentioned, another possible explanation for the difference in results is that inactivated native NiV and HeV were used in the current study, while the other studies used recombinant NiV/HeV glycoproteins. Compared with the use of recombinant proteins, the use of wild-type viruses is more relevant, especially for the development of diagnostic tests. Another possibility is that the viral inactivation process may have altered the conformation of the NiV/HeV G protein on the native virus, yielding different results from recombinant protein studies. In any case, ephrin B2 was chosen as the ligand for henipavirus detection in this study. Sandwich ELISA confirms capture of henipavirus-G by ephrin B2, consistent with previous findings that ephrin B2 binds to henipavirus-G [37].

For henipavirus detection, RT-PCR is recommended as the most sensitive method for the diagnosis of active NiV infections [38]. Other detection methods include virus isolation and immunohistochemistry. All these methods require trained personnel, laboratory equipment, and a relatively long time to obtain results. Antigen detection ELISA can be considered an alternative diagnostic approach, and NiV antigen-capture ELISAs have been reported previously [39,40]. However, both previously reported tests used polyclonal anti-NiV antibodies, which have several disadvantages, including non-specific interaction with unrelated antigens and batch-to-batch inconsistency. Therefore, testing with polyclonal antibodies can be difficult to standardize. Conversely, recombinant proteins have higher levels of consistency between batches, confirmed specificity, and a guaranteed long-term supply. Therefore, instead of using a polyclonal antibody, we used recombinant ephrin B2 as the capture ligand in the ELISA and two mAbs, F20NiV-65 for NiV and F27NiV-34 for NiV and HeV. Our results demonstrated that ephrin B2 can capture both NiV and HeV in ELISAs. Since both NiV and HeV cause severe disease in animals and humans and have similar clinical symptoms, it is necessary to have a method to differentiate the two for early diagnosis and outbreak control. Our newly developed ELISA accomplishes this differentiation by using ephrin B2 in combination with two different mAbs.

Given that NiV outbreaks often occur in rural and remote areas, simple and fast henipavirus detection and diagnosis methods, such as point-of-care (POC) testing, need to be deployed to the field. Compared to laboratory-based diagnosis, POC tests have less onerous requirements for personnel training and equipment. Therefore, they can be carried out more easily in the field or outbreak site. The LFI strip test is by far one of the most successful analytical platforms to perform the on-site detection of target substances [41]. Currently, there is no LFI strip test available for henipavirus detection. In this study, using the commercially available gRAD strips, biotin-conjugated ephrin B2, and gold conjugated mAb F20NiV-60, we developed a prototype of a strip test for NiV detection. The strip test is NiV-specific, has a very weak positive signal for HeV, and does not cross-react with the negative control FMDV. Interestingly, mAb F27NiV-34 failed to detect henipaviruses in the LFI strip test, despite detecting both NiV and HeV when paired with ephrin B2 in an ELISA. One possible explanation is that during the ELISA the ephrin B2/virus/antibody complex forms on the surface of polystyrene plates, whereas during the prototype strip test, the reaction takes place in liquid. The different binding formats may produce different steric hindrances, leading to differences in ephrin B2/virus/antibody binding.

## 5. Conclusions

The Henipavirus receptor ephrin B2 can be used as a capture ligand for NiV and HeV detection in ELISA and LFI strip tests. Ephrin B2 shows a similar ability to capture both NiV and HeV compared to polyclonal anti-NiV-G in antigen detection ELISA. This finding provides valuable information for the future development of immunoassays for accurate and rapid detection of NiV and HeV. Since the main goal of this report is to approve the concept that recombinant ephrinB2 can be used as a capture ligand for the detection of Henipaviruses, the ELISA and LFI strip test developed in this study are both prototypes.

Currently, only inactivated viruses are used. In the future, if possible, both virus detection methods will be validated using a live virus, samples from experimentally infected animals, and samples from the field. The diagnostic specificity and sensitivity of ELISA and lateral flow strip tests have not been established, but they will be determined later. The sensitivity of both tests can be further improved by titration of capture and detection reagents. While neither virus detection method will be as sensitive as RT-PCR, it is hoped that the sensitivity of the strip test will be comparable to the virus detection ELISA.

## Data Availability

The data presented in this study are available on request from the corresponding author.

## Supplementary Materials

The following supporting information can be downloaded at: https://www.mdpi.com/article/10.3390/v14081657/s1, Figure S1: Western blot analysis of recombinant Nipah glycoprotein (*GP*); Figure S2: Western blot analysis of recombinant human ephrin B2.
