# Supplementary material for: Detection of Nipah and Hendra Viruses Using Recombinant Human Ephrin B2 Capture Virus in Immunoassays
Source: Viruses. 2022 Jul 28;14(8):1657. doi: 10.3390/v14081657 (PMC9415732; doi:10.3390/v14081657)
Supplement: Supplementary file 1 [file viruses-14-01657-s001.zip › viruses-1792930-supplementary.pdf]

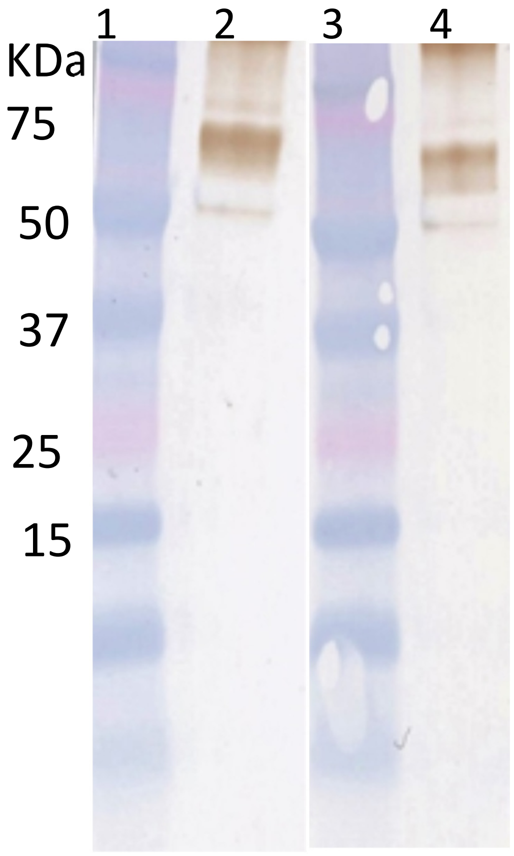

Figure S1. Western blot analysis of recombinant Nipah glycoprotein. NiV glycoprotein (G) was separated on a 10% SDS-PAGE and the protein was transferred to a NC membrane. The NiV-G was detected with HRP-conjugated anti-his mAb and polyclonal rabbit anti-NiV-G, followed by incubation with HRP–conjugated anti-rabbit for 1 hour at room temperature. The blots were developed using 3, 3'–Diaminobenzidine. Lane 1, 3: molecular weight marker, and lane 2, 4: recombinant NiV-G with anti his-tag and anti-NiV-G, respectively.

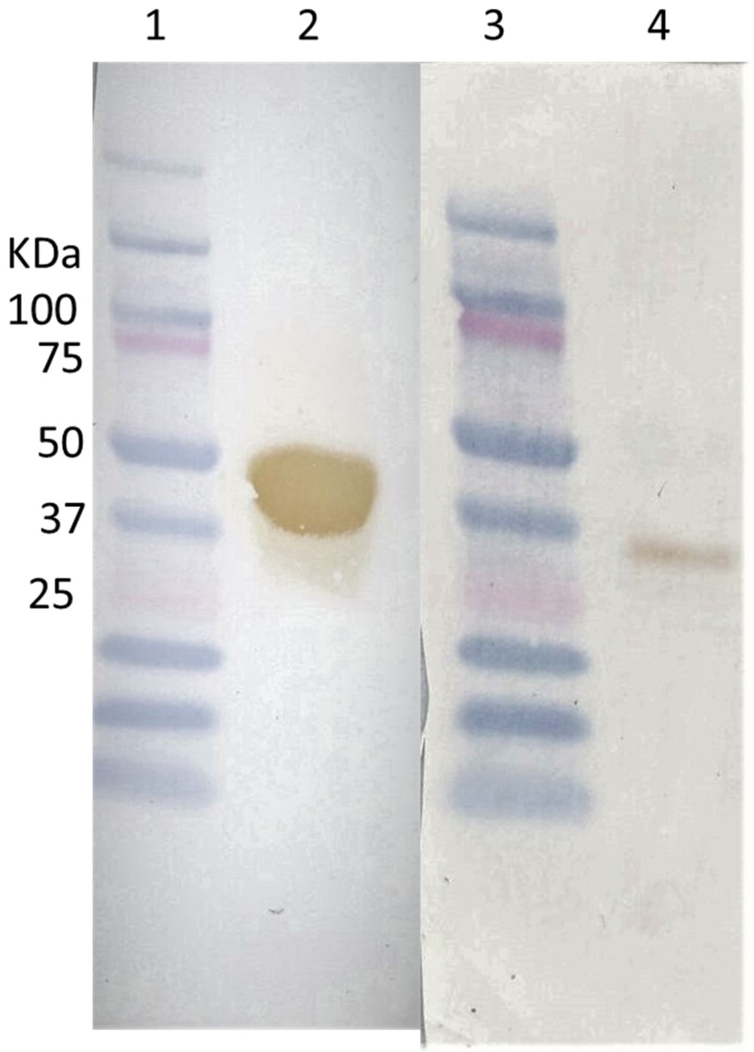

Figure S2. Western blot analysis of recombinant human ephrin B2. Recombinant human ephrin B2 was separated on a 10% SDS-PAGE and the protein was transferred to the NC membrane. The ephrin B2 was detected with (a) HRP-conjugated mAb to His-tag, and (b) monoclonal anti-ephrin B2 antibody, followed by incubation with HRP–conjugated anti-mouse IgG for 1 hour at room temperature. Both blots were developed using 3, 3'–Diaminobenzidine (Sigma–Aldrich, St. Lucia, USA). Lane 1, 3: molecular weight marker, and lane 2, 4: recombinant ephrin B2 with anti his-tag and anti-Ephrin B2, respectively.
